# Ignition temperature and explosion pressure of suspended coal dust cloud under different conditions and suppression characteristics

**DOI:** 10.1038/s41598-023-42117-x

**Published:** 2023-09-08

**Authors:** Tianqi Liu, Xiangzhen Mu, Xingchen Wu, Ruiheng Jia, Jining Xie, Zhongyi Gao

**Affiliations:** School of Safety Engineering, Shenyang Aerospace University, Shenyang, 110136 Liaoning China

**Keywords:** Chemistry, Energy science and technology

## Abstract

The ignition and explosion processes of suspended coal dust clouds and their suppression characteristics are important aspects of dust prevention and control. To understand the ignition temperature and explosion pressure of coal dust clouds, as well as the inhibitory effect of explosion suppressants, experimental tests are conducted. The study found that during the ignition process of coal dust clouds, the optimal dust spray pressure is 20 kPa, because coal dust clouds are more likely to ignite under this condition. When the mass concentration of coal dust cloud is 500 g m^−3^, the maximum pressure and maximum pressure rise rate are both the highest. When Al(OH)_3_ is mixed with coal dust and the mass percentage is 60%, the coal dust cloud can still be ignited. When KH_2_PO_4_ is mixed with coal dust, the upper limit of the test temperature is reached when the percentage of mixture is 55%. When NH_4_H_2_PO_4_ is mixed with coal dust and the mass percentage is greater than 40%, the coal dust cloud can’t be ignited anymore. The suppression effect of mixing Al(OH)_3_ and NH_4_H_2_PO_4_ is not as good as that of mixing KH_2_PO_4_ and NH_4_H_2_PO_4_.

## Introduction

Coal dust can be suspended in mines, causing serious explosion accidents. Explosion of suspended coal dust clouds can bring devastating disasters to mines^1^. The most important condition for coal dust explosion is that the coal dust cloud is ignited at a certain temperature or energy. If the ignition temperature or energy cannot ignite the coal dust cloud, then the explosion cannot occur^2,3^. So, ignition temperature is a key factor causing coal dust cloud explosions. After a coal dust cloud explosion, a huge pressure wave will form, providing conditions for secondary or even multiple explosions, making it highly noteworthy^4,5^. Against the backdrop of the great harm caused by coal dust cloud explosions, research on the suppression of coal dust explosions has attracted increasing attention from scholars^6,7^. This article focuses on the ignition temperature and pressure characteristics of suspended coal dust clouds under different conditions, as well as the inhibitory effect of explosion suppressants on ignition temperature and pressure.

Scholars have achieved some results in the study of the ignition characteristics and explosion pressure characteristics of dust clouds, mainly focusing on the ignition temperature and energy under different conditions, as well as the propagation process of explosion pressure^8–10^. The research on the mechanism of dust explosion has also been widely discussed. Although the micro mechanism of dust explosion has not been fully understood yet, by comparing the mechanism of dust explosion with that of gas explosion, the micro process of dust explosion has become increasingly familiar to people^11–15^. Related studies have found that different particle sizes, dust cloud mass concentrations, and ignition temperatures have important effects on explosion pressure^16–21^. In addition, the inhibitory effects of different explosion suppressants on dust explosions have also been studied. Scholars have found that the decomposition process of dust particles under heating conditions is related to many factors. The spatial scale of the explosion can affect the pressure of the explosion, and the type and dosage of explosion suppressants can also affect the effectiveness of explosion suppression.

From the perspective of explosion suppression mechanism, the mechanism of using explosion suppressants to suppress dust explosions mainly includes physical suppression and chemical suppression. Some studies have also found that combining physical and chemical explosion suppression methods may result in better suppression effects^22–25^. Regardless of the type of explosion suppression method, scholars prefer to use inexpensive and effective explosion suppressants. On the basis of continuous research on the effectiveness of explosion suppression, research results on the use of mixed explosion suppressants continue to emerge, which will be an important achievement in dust explosion suppression research^26–28^. The selection of explosion suppressants can often refer to fire extinguishing agents. Industrial fire extinguishing agents have excellent fire extinguishing effects, and they also have the same effect in suppressing dust explosions^29,30^. At present, although some achievements have been made in research on dust explosion suppression, research in this area is still ongoing.

From the above analysis, it can be seen that research on the ignition temperature and pressure characteristics of dust has made certain progress. The changes in ignition temperature under different conditions still need further discussion, and the inhibitory effects of different explosion suppressants on dust explosions have received widespread attention. In the previous research, the author discussed the flame propagation process of coal dust explosion and the influence of ignition energy on coal dust explosion, and used computational fluid dynamics to simulate the propagation process of coal dust explosion^31–35^. The author's previous research results provide an important foundation for the research of this article. In this article, the author takes suspended coal dust clouds as the research object, analyzes the characteristics of coal dust cloud ignition temperature and pressure changes under different conditions, and then uses different explosion suppressants to study their suppression effects on ignition temperature and explosion pressure. The research results will be of great significance for understanding the explosion characteristics and suppression process of coal dust clouds.

## Experimental equipments and samples

### Experimental equipments

**Figure 1:**
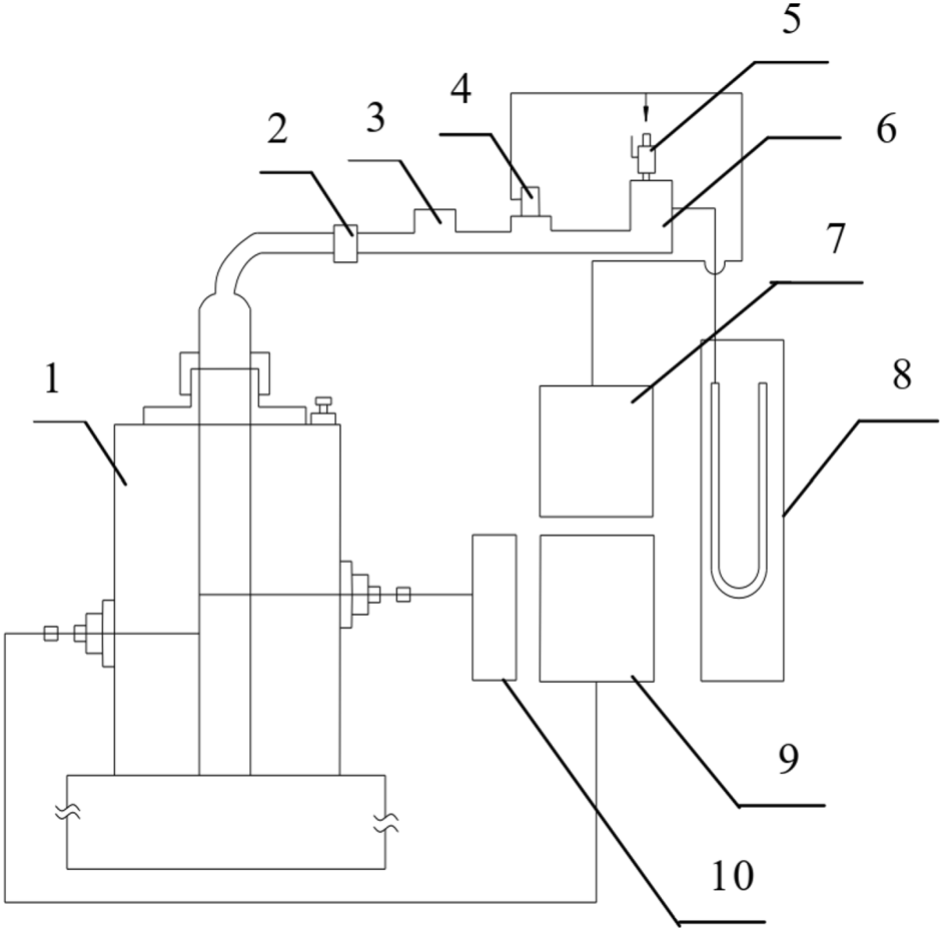
Experimental equipment for minimum ignition temperature of coal dust clouds. 1 heating furnace; 2 connector; 3 dust container; 4 electromagnetic valve; 5 gate valve; 6 gas tank; 7 power supply; 8 U tube; 9 temperature controller; 10 temperature recorder.

The experiments in this article include testing the minimum ignition temperature of coal dust clouds and measuring the explosion pressure of coal dust clouds. The experimental equipment for testing the minimum ignition temperature of coal dust clouds is shown in Fig. 1. It mainly consists of the heating furnace, the connector, and the dust container, etc. During the testing process, the minimum ignition temperature of suspended coal dust clouds under different conditions can be obtained. Similarly, in explosion suppression experiments, coal dust and explosion suppressants can also be mixed and tested to obtain the effect of different explosion suppressants on the minimum ignition temperature of coal dust clouds.

**Figure 2:**
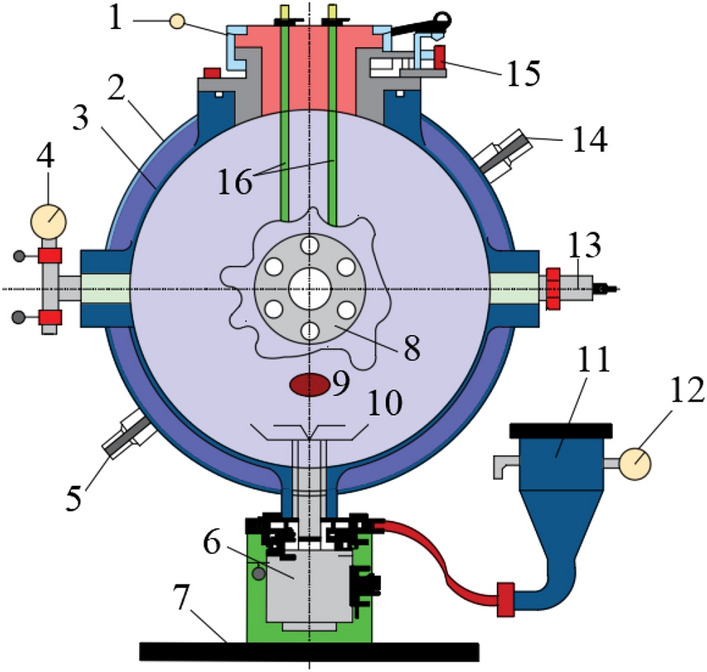
Structure diagram of explosive device. 1 sealing cap; 2 outer side of mezzanine; 3 inside of mezzanine; 4 vacuum gauge; 5 outlet of water; 6 mechanical two-way valve; 7 base; 8 observation window; 9 vacuum hole; 10 dispersion valve; 11 storage tank; 12 pressure gauge; 13 pressure sensor; 14 inlet of water; 15 limit switch; 16 ignition rod.

The experimental equipment for testing the explosion pressure of suspended coal dust clouds is shown in Fig. 2. It is mainly composed of the sealing cap, the dispersion valve, the storage tank, and the ignition rod, etc. The suspended coal dust cloud will explode instantly in the equipment, and the pressure curve obtained from the explosion will display the maximum pressure *P*_max_ and the maximum pressure rise rate (d*P*/d*t*)_max_. In coal dust explosion experiments, the ignition energy used is usually 10 kJ, because for coal dust clouds, this order of magnitude of ignition energy can successfully ignite coal dust clouds. After mixing the coal dust and the explosion suppressants, the explosion experiments can be conducted to obtain the effects of different explosion suppressants on explosion pressure.

### Experimental samples

**Figure 3:**
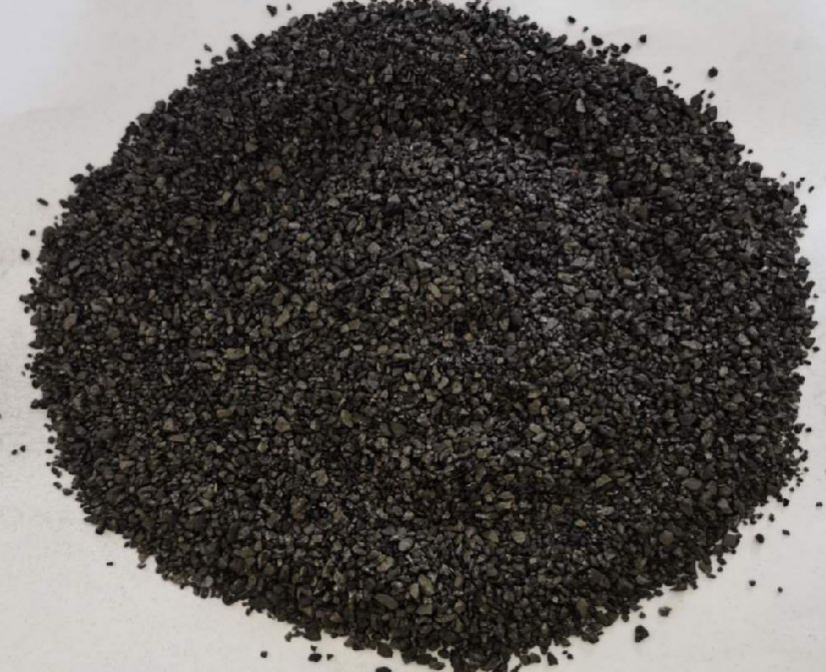
Selected experimental coal sample.

The coal dust samples used in the article were selected from mining areas in northwest China. Figure 3 shows the particles of the experimental coal sample. Usually, micron and nanoscale coal dust carries a significant risk of ignition and explosion. The particle size of the coal dust samples used in this article is in the micrometer scale. The composition of coal samples was tested using industrial and elemental analyzers, and the industrial and elemental analysis results of the coal samples obtained are shown in Table 1. From the industrial analysis results, it can be seen that the fixed carbon content of the coal sample is the largest, indicating that it is the main component of the coal sample, followed by volatile matter. Volatile matter is the combustible gas that will evaporate after the coal sample is heated. The proportion of fixed carbon and volatile matter exceeds 80%, both of which are the main components of coal samples. From the elemental analysis results, it can be seen that carbon is the main element in the coal sample, followed by oxygen, which are also the main elements of organic matter.

**Table 1:** Proximate analysis and ultimate analysis of coal dust sample.

Proximate analysis (%)				Ultimate analysis (%)			
*M*	*A*	*V*	*FC*	C	H	O	N
6.93	9.88	33.95	49.24	59.37	4.62	25.93	10.08

*M*: moisture; *A*: ash; *V*: volatile; *FC*: fixed carbon.

**Figure 4:**
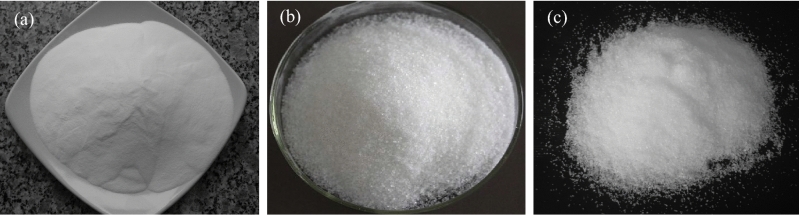
Selected explosion suppressants: (**a**) Al(OH)_3_, (**b**) KH_2_PO_4_, and (**c**) NH_4_H_2_PO_4_.

In the experiment of suppressing the ignition and explosion of suspended coal dust clouds, the selected suppressants are Al(OH)_3_, KH_2_PO_4_, and NH_4_H_2_PO_4_. They are all typical components of fire extinguishing agents. After mixing the explosion suppressant with coal dust for ignition and explosion experiments, the inhibitory effect of the explosion suppressant on coal dust ignition and explosion can be obtained. Three types of explosion suppressants are shown in Fig. 4, all of which are white powder like particles. Table 2 shows the physical properties of three types of explosion suppressants. Al(OH)_3_ is insoluble in water, while KH_2_PO_4_ and NH_4_H_2_PO_4_ are soluble in water. The explosion suppressant with the highest molecular weight is KH_2_PO_4_, and the explosion suppressant with the highest density is Al(OH)_3_.

**Table 2:** Physical properties of explosion suppressants.

Chemical formula	Molecular weight	Density (g/cm^3^)	Water-solubility
Al(OH)_3_	78.004	2.40	Insoluble
KH_2_PO_4_	136.086	2.338	Soluble
NH_4_H_2_PO_4_	115.026	1.02	Soluble

## Results and discussion

### Ignition temperature and explosion pressure of suspended coal dust clouds

#### Minimum ignition temperature of suspended coal dust cloud under different conditions

The particle size of the coal samples used in the experiment is less than 75 μm. The minimum ignition temperature of coal dust cloud under the condition of spraying dust pressure of 20 kPa is 983 K, as shown in Table 3. In the confined space of the experimental equipment, at a temperature of 983 K, suspended coal dust clouds are ignited, which is the critical temperature at which coal dust clouds can be ignited. Below this temperature, coal dust clouds cannot be ignited. This test data can provide a basis for understanding the combustion and explosion characteristics of coal dust.

**Table 3:** Minimum ignition temperature of suspended coal dust cloud.

Coal sample	Ignition condition		Minimum ignition temperature (K)
	Particle size (μm)	Dust spray pressure (kPa)	
Lean coal	< 75	20	983

**Figure 5:**
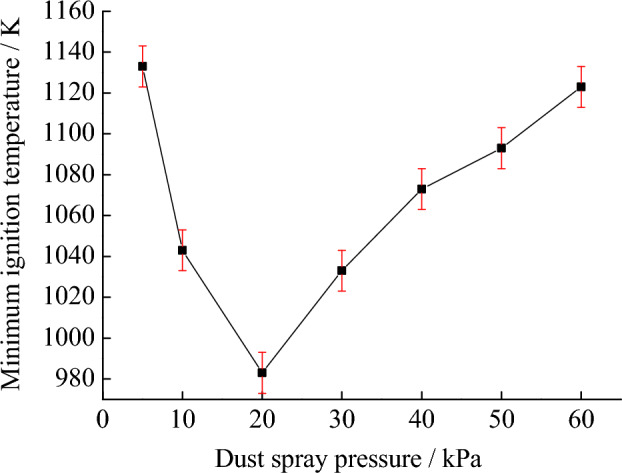
Minimum ignition temperature under different dust spray pressure conditions.

On this basis, the minimum ignition temperature of coal dust clouds can be tested under different spray pressure conditions. The test results are shown in Fig. 5. It can be obtained that when the dust spray pressure is greater than 20 kPa, the minimum ignition temperature of the suspended coal dust cloud increases. When the dust spray pressure is 60 kPa, the minimum ignition temperature is 1123 K. When the dust spray pressure is less than 20 kPa, the minimum ignition temperature of the suspended coal dust cloud increases. When the dust spray pressure is 5 kPa, the minimum ignition temperature is 1133 K. These results indicate that the optimal dust spray pressure is 20 kPa. Under this condition, coal dust clouds are more likely to ignite.

**Figure 6:**
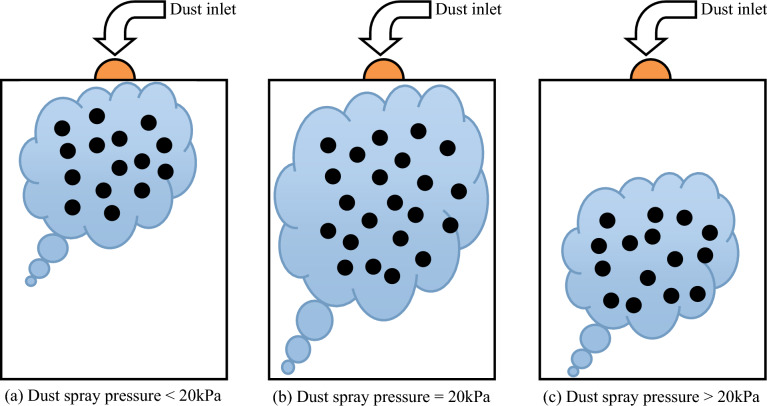
Effect of dust spray pressure on ignition of suspended coal dust clouds.

As shown in Fig. 6, the effect of dust spray pressure on the diffusion of coal dust cloud particles in the ignition space is presented. In the experimental equipment, when the dust spray pressure is 20 kPa, the coal dust cloud diffuses more evenly, and more coal dust particles move into the ignition space, forming a suspended coal dust cloud. Due to the heat exchange and transfer between coal dust particles, the coal dust cloud under this condition is more easily ignited. If the spraying pressure is less than 20 kPa, the driving force obtained by coal dust particles is significantly insufficient, and the number of coal dust particles that can enter the ignition space is greatly reduced. The particles are concentrated in the upper part of the space, which weakens the heat exchange between particles. To be ignited, higher temperatures are required. If the spraying pressure is greater than 20 kPa, the power to drive coal dust particles into the ignition space is relatively high. Under the action of gravity, more coal dust will be concentrated below the interior of the ignition space, which is not conducive to the release and exchange of heat from coal dust particles, and the temperature required for ignition will be higher.

#### Explosion pressure of suspended coal dust clouds under different conditions

In the explosion pressure test experiment, the dust spray pressure is 2 MPa, the ignition delay time is 0.1 s, and the ignition energy is 10 kJ. According to the experimental results, the maximum pressure of suspended coal dust cloud explosion is 0.78 MPa, and the maximum pressure rise rate is 73.27 MPa s^−1^, the results are shown in Table 4. Under normal circumstances, the standard atmospheric pressure is 0.1 MPa, and an explosion produces a maximum pressure of 0.78 MPa. The required coal dust is only 10 g. This explosion has a great power, and the concentration of coal dust clouds in the explosion space can reach 500 g m^−3^. This concentration condition is very favorable for the development of the explosion.

**Table 4:** Test results of coal dust cloud explosion pressure.

Dust spray pressure (MPa)	Ignition delay time (s)	Ignition energy (kJ)	Explosion pressure	
			*P*_max_ (MPa)	(d*P*/d*t*)_max_ (MPa s^−1^)
2	0.1	10	0.78	73.27

**Figure 7:**
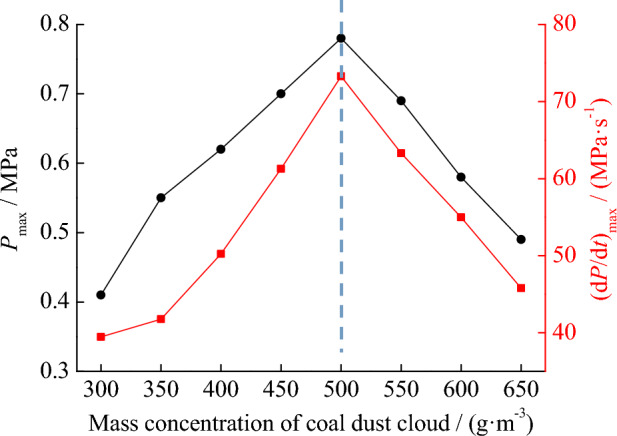
Relationship between mass concentration of coal dust clouds and explosion pressure.

In the explosion pressure experiment mentioned above, the mass of coal dust used is 10 g. The change in coal dust quality will affect the concentration of suspended coal dust clouds in the explosion space. The concentration of coal dust clouds can have a significant impact on explosion pressure. Therefore, in order to study the effect of coal dust cloud concentration on explosion pressure, experiments can be conducted continuously by changing the amount of coal dust used. The relationship between the mass concentration of coal dust cloud obtained from the test and the explosion pressure is shown in Fig. 7. It can be clearly seen that when the mass concentration of coal dust cloud is 500 g m^−3^, the maximum pressure and maximum pressure rise rate are both the highest, with values of 0.78 MPa and 73.27 MPa s^−1^, respectively. When the mass concentration of coal dust cloud is less than or greater than 500 g m^−3^, the maximum pressure and the maximum rate of pressure rise will decrease. This is because if the mass concentration of coal dust clouds is too small, the coal dust particles that release heat are insufficient. If the mass concentration of coal dust clouds is too high, the oxygen required for the explosion will be insufficient. There is a dynamic equilibrium between coal dust particles and oxygen. When the mass concentration of coal dust cloud is 500 g m^−3^, the suspended coal dust cloud reaches this equilibrium state, and the explosion pressure is also the maximum.

### Inhibitory effect of explosion suppressants on ignition temperature and explosion pressure of coal dust clouds

#### Inhibitory effect of explosion suppressants on ignition temperature of coal dust clouds

In the previous text, the minimum ignition temperature of suspended coal dust clouds under different conditions was obtained. Next, use explosion suppressants Al(OH)_3_, KH_2_PO_4_, and NH_4_H_2_PO_4_ to study their suppression characteristics on the minimum ignition temperature of coal dust clouds. When the dust spray pressure is 20 kPa, the minimum ignition temperature of the coal dust cloud is 983 K. Under this condition, mix different explosion suppressants with coal dust particles separately, and then test the minimum ignition temperature of the mixture. The upper limit for experimental equipment testing is 1273 K. The mass percentage of the explosion suppressant mixed with coal dust is *p*. The dust particle size of the explosion suppressant is also 0–75 μm. Table 5 shows the minimum ignition temperature of coal dust clouds under different mass percentages of suppressants mixed with coal dust.

**Table 5:** Inhibitory effect of three explosion suppressants on minimum ignition temperature of coal dust clouds.

*p* (%)	Minimum ignition temperature (K)		
	Al(OH)_3_ + coal dust	KH_2_PO_4_ + coal dust	NH_4_H_2_PO_4_ + coal dust
0	983	983	983
5	993	1003	1023
10	1023	1033	1053
15	1033	1053	1093
20	1043	1083	1123
25	1053	1093	1163
30	1073	1113	1193
35	1093	1143	1233
40	1103	1173	1273
45	1133	1193	–
50	1153	1233	–
55	1183	1273	–
60	1213	–	–

**Figure 8:**
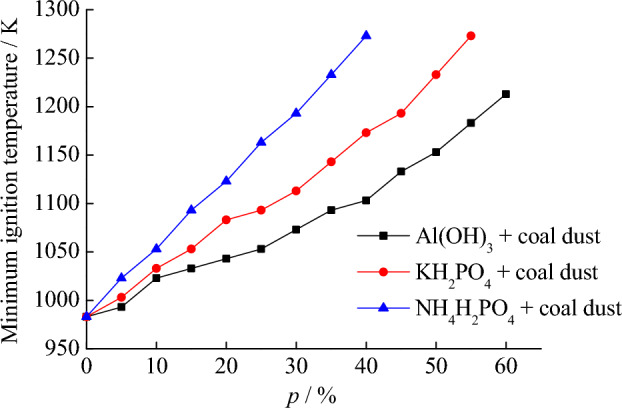
Inhibitory effect of different explosion suppressants on minimum ignition temperature of coal dust clouds.

As shown in Table 5, the minimum ignition temperature after mixing different explosion suppressants and coal dust significantly increases. When Al(OH)_3_ is mixed with coal dust and the mass percentage is 60%, the minimum ignition temperature of the coal dust cloud is 1213 K. Under this condition, the coal dust cloud can still be ignited. When KH_2_PO_4_ is mixed with coal dust, the upper limit of the test temperature is just reached when the percentage of mixture is 55%. When NH_4_H_2_PO_4_ is mixed with coal dust, the upper limit of the test temperature is also reached when the percentage of mixture is 40%. Based on the above results, the inhibitory effects of three explosion suppressants can be compared, with NH_4_H_2_PO_4_ having the strongest inhibitory effect, KH_2_PO_4_ having the second strongest inhibitory effect, and Al(OH)_3_ having the weakest inhibitory effect. Figure 8 is drawn to compare the inhibitory effects of explosion suppressants. It can also be found that the inhibitory effect of NH_4_H_2_PO_4_ is the greatest. When *p* is 40%, within the upper limit range of the test temperature, the ignition of coal dust clouds can be completely suppressed.

The following analyzes the impact of explosion suppressants on the ignition process of coal dust clouds from the perspective of ignition suppression mechanism. After mixing Al(OH)_3_ with coal dust, under heating conditions, Al(OH)_3_ will undergo the following chemical reaction: Al(OH)_3_ → Al_2_O_3_ + H_2_O. The generated Al_2_O_3_ will prevent the coal dust particles from releasing heat outward, while the generated H_2_O will evaporate and absorb heat. When heated to 673 K, KH_2_PO_4_ generates KPO_3_ and H_2_O. KPO_3_ will block the heat exchange of coal dust particles, and H_2_O will be evaporated to absorb heat. Under the heating conditions of NH_4_H_2_PO_4_, P_2_O_5_, H_2_O, and NH_3_ are generated. P_2_O_5_ is a solid product that can prevent heat transfer and absorb space heat. H_2_O is a liquid product that can evaporate and absorb heat. NH_3_ is a gas product that can dilute oxygen. Due to the fact that NH_4_H_2_PO_4_ generates more types of products after being heated, it plays a greater role in suppressing the ignition of coal dust clouds.

#### Inhibitory effect of explosion suppressants on explosion pressure of coal dust clouds

On the basis of the previous discussion on the suppression effect of Al(OH)_3_, KH_2_PO_4_, and NH_4_H_2_PO_4_ on the minimum ignition temperature of suspended coal dust cloud, although the inhibitory effect of different explosion suppressants has been obtained, due to the relatively high cost of NH_4_H_2_PO_4_ dust, it is difficult to use NH_4_H_2_PO_4_ dust alone to suppress the explosion of coal dust in industry. Therefore, in order to reduce the cost, in this part, the mixing of different suppression dust according to different schemes is considered, and the mixed suppression dust is used to study the explosion pressure suppression effect.

The specific explosion test scheme is as follows: the mass of the coal dust sample is still 10 g, of which 100% of the particle size is 0 ~ 75 μm, because the micron sized coal dust particles are explosive, which is convenient to observe the suppression effect. The dust particle size of the explosion suppressant is also 0–75 μm.

**Figure 9:**
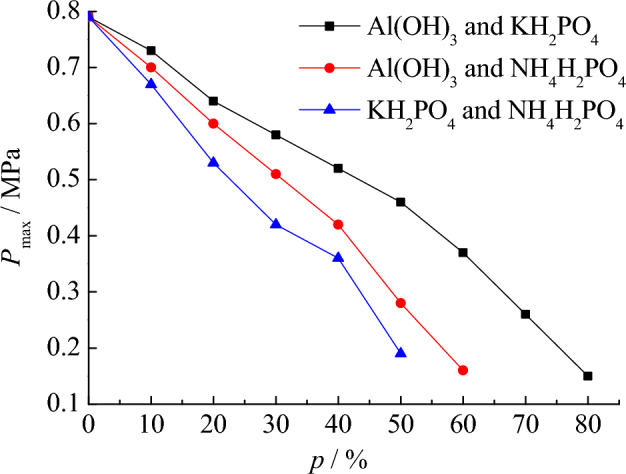
Suppression of mixed suppression dust on *P*_max_.

**Figure 10:**
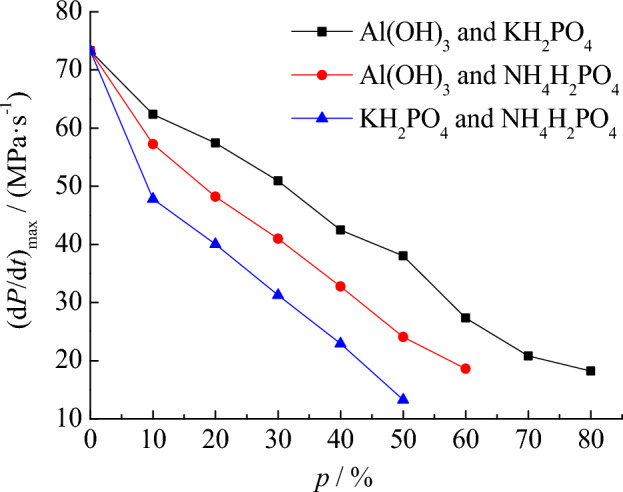
Suppression of mixed suppression dust on (d*P*/d*t*)_max_.

In addition, among the three types of explosion suppression dust Al(OH)_3_, KH_2_PO_4_, and NH_4_H_2_PO_4_, since NH_4_H_2_PO_4_ has the greatest suppression effect, we should focus on the results of the explosion suppression experiment with the participation of NH_4_H_2_PO_4_. There are three plans to mix explosive suppression dust, the first is to mix Al(OH)_3_ and KH_2_PO_4_, the second is to mix Al(OH)_3_ and NH_4_H_2_PO_4_, and the third is to mix KH_2_PO_4_ and NH_4_H_2_PO_4_. In these three schemes, the mass percentage of different two types of explosion suppression dust is 50% for both. On this basis, the explosion suppression data obtained from the test are shown in Table 6. Using the data in Table 6, the explosion pressure curve under the condition of mixed explosion suppression dust is drawn, and the results are shown in Figs. 9 and 10.

**Table 6:** Suppression of mixed explosion suppression dust on suspended coal dust explosion pressure.

*p* (%)	Al(OH)_3_ and KH_2_PO_4_		Al(OH)_3_ and NH_4_H_2_PO_4_		KH_2_PO_4_ and NH_4_H_2_PO_4_	
	*P*_max_ (MPa)	(d*P*/d*t*)_max_ (MPa s^−1^)	*P*_max_ (MPa)	(d*P*/d*t*)_max_ (MPa s^−1^)	*P*_max_ (MPa)	(d*P*/d*t*)_max_ (MPa s^−1^)
0	0.78	73.27	0.78	73.27	0.78	73.27
10	0.73	62.36	0.70	57.26	0.67	48.69
20	0.64	57.43	0.60	48.19	0.53	41.92
30	0.58	50.93	0.51	40.98	0.42	32.55
40	0.52	42.47	0.42	32.77	0.36	23.28
50	0.46	38.04	0.28	24.08	0.19	14.73
60	0.37	27.35	0.16	18.62	–	–
70	0.26	20.82	–	–	–	–
80	0.15	18.20	–	–	–	–
90	–	–	–	–	–	–

By comparing the explosion suppression effect of mixed explosion suppression dust and single explosion suppression dust, the advantages of mixed explosion suppression dust can be better understood. When using a single type of explosion suppression dust, the best effect of explosion suppression dust is NH_4_H_2_PO_4_, followed by KH_2_PO_4_, and the worst is Al(OH)_3_. As shown in Figs. 9 and 10, under the condition of using mixed explosion suppression dust, the explosion suppression effect of mixing KH_2_PO_4_ and NH_4_H_2_PO_4_ in 1:1 ratio is worse than that of using NH_4_H_2_PO_4_ dust alone, because when KH_2_PO_4_ and NH_4_H_2_PO_4_ are mixed and the mass percentage of mixed explosion suppression dust is 50%, the maximum explosion pressure and the maximum pressure rise rate are 0.19 MPa and 14.73 MPa s^−1^, respectively, which is larger than the data of using NH_4_H_2_PO_4_ dust alone for explosion suppression. It shows that reducing the proportion of NH_4_H_2_PO_4_ in the explosion suppression dust will reduce the explosion suppression effect, but it will greatly save the cost to a certain extent. When using KH_2_PO_4_ and NH_4_H_2_PO_4_ as the mixed explosion suppression dust, once the mass percentage of suppression dust mixed into coal dust is 60%, the explosion can also be prevented. Therefore, it is proved that selecting the method of KH_2_PO_4_ and NH_4_H_2_PO_4_ mixing instead of using NH_4_H_2_PO_4_ alone can also control the explosion to a certain extent. This analysis result is of great significance for explosion suppression research, because the explosion suppression effect of NH_4_H_2_PO_4_ is indeed better than that of KH_2_PO_4_, reducing the percentage of NH_4_H_2_PO_4_ will certainly reduce the explosion suppression effect.

Further analysis shows that the explosion suppression effect of mixing Al(OH)_3_ and NH_4_H_2_PO_4_ is not as good as that of mixing KH_2_PO_4_ and NH_4_H_2_PO_4_. When Al(OH)_3_ and NH_4_H_2_PO_4_ are mixed and the mass percentage of the suppression dust mixed into coal dust is 60%, the explosion is still not completely suppressed. The maximum pressure and the maximum pressure rise rate of the explosion are 0.16 MPa and 18.62 MPa s^−1^, respectively. When the mass percentage of suppression dust increases to 70%, the explosion will not occur again. Therefore, in terms of the explosion suppression effect, the explosion suppression effect of mixture Al(OH)_3_ and NH_4_H_2_PO_4_ is not as good as that of mixture KH_2_PO_4_ and NH_4_H_2_PO_4_.

Finally, the explosion suppression characteristics under the condition of mixing Al(OH)_3_ and KH_2_PO_4_ are discussed. From the test data, it can be seen that the explosion suppression effect after mixing Al(OH)_3_ and KH_2_PO_4_ is the worst among the three types of mixing schemes. When the mass percentage of suppression dust mixed into coal dust is 80%, the maximum explosion pressure and the maximum pressure rise rate are respectively 0.15 MPa and 18.20 MPa s^−1^. The explosion does not occur until the mass percentage of the explosion suppression dust is increased to 90%, which is not ideal by comparison. The above analysis results are of great significance for understanding the suppression effect of coal dust cloud explosion pressure under different mixed conditions of explosion suppressants.

## Conclusions

In this article, suspended coal dust clouds are selected as the research object, and the ignition temperature and pressure characteristics of coal dust clouds under different conditions are analyzed. The inhibitory effects of different explosion suppressants on the ignition and explosion of coal dust clouds are obtained. The specific conclusions are as follows.

By testing the ignition temperature and explosion pressure of coal dust clouds under different conditions, it is found that the optimal dust spray pressure is 20 kPa, because coal dust clouds are more likely to ignite under this condition. When the mass concentration of coal dust cloud is 500 g m^−3^, the maximum pressure and maximum pressure rise rate are both the highest, indicating that there is a dynamic equilibrium between coal dust particles and oxygen.

After mixing different explosion suppressants with coal dust and testing the ignition temperature, it is found that when Al(OH)_3_ is mixed with coal dust and the mass percentage is 60%, the coal dust cloud can still be ignited. When KH_2_PO_4_ is mixed with coal dust, the upper limit of the test temperature is reached when the percentage of mixture is 55%. When NH_4_H_2_PO_4_ is mixed with coal dust and the mass percentage is greater than 40%, the coal dust cloud can’t be ignited anymore.

By analyzing the pressure suppression characteristics of coal dust cloud explosion under different mixed conditions of explosion suppressants, it is found that the suppression effect of mixing Al(OH)_3_ and NH_4_H_2_PO_4_ is not as good as that of mixing KH_2_PO_4_ and NH_4_H_2_PO_4_. When Al(OH)_3_ and NH_4_H_2_PO_4_ are mixed and the mass percentage is 60%, the explosion is still not completely suppressed.

## Data Availability

All data generated or analysed during this study are included in this published article.
